# Efficacy and safety of *Tripterygium* glycosides as an add-on treatment in adults with chronic urticaria: a systematic review and meta-analysis

**DOI:** 10.1080/13880209.2023.2169468

**Published:** 2023-01-24

**Authors:** Ming Li, Yan Li, Lujing Xiang

**Affiliations:** Department of Dermatology, Beijing Friendship Hospital, Capital Medical University, Beijing, China

**Keywords:** Randomized controlled trials, traditional Chinese medicine, effectiveness, adverse event

## Abstract

**Context:**

*Tripterygium* glycosides (TG), a traditional Chinese medicine, has been used to treat chronic urticaria (CU) in China, and the evidence of TG for CU needs to be updated thoroughly.

**Objective:**

To systematically evaluate the efficacy and safety of TG combined with H1-antihistamine (H1-AH) in adults with CU.

**Methods:**

Eligible randomized controlled trials were searched in eight databases until May 31, 2022, including CNKI, WanFang, VIP, SinoMed, PubMed, Cochrane Library, Embase, and Web of Science. The search terms included urticaria, *Tripterygium*, Lei Gong Teng, and Leigongteng. Rev Man 5.3 and Stata 12.0 were used for statistical analysis.

**Results:**

A total of 27 studies with 2788 patients were included. The pooled results showed that TG plus H1-AH was superior to H1-AH alone in cure rate (RR = 1.37, 95% CI = 1.15 to 1.63, *p* = 0.0003), total efficacy rate (RR = 1.40, 95% CI = 1.30 to 1.50, *p* < 0.00001), pruritus (MD = −0.32, 95% CI = −0.54 to −0.11, *p* = 0.003), wheal number (MD = −0.31, 95% CI = −0.55 to −0.07, *p* = 0.01), wheal size (MD = −0.32, 95% CI = −0.46 to −0.19, *p* < 0.00001), and the serum level of immunoglobulin E (SMD = −1.39, 95% CI = −2.42 to −0.36, *p* = 0.008). Moreover, adverse events between two groups were mild, and their incidences were not significantly different.

**Conclusions:**

The combination of TG and H1-AH is a promising and safe treatment for adults with refractory CU. Further high-quality studies are needed to confirm the evidence.

## Introduction

Chronic urticaria (CU) is a common inflammatory skin disease characterized by the recurrence of wheals, angioedema, or both for more than 6 weeks (He et al. 2021). Within the worldwide prevalence of CU ranging from 0.1% to 3.4% (Fricke et al. 2020), the prevalence of adults with CU in China is estimated at 2.6%, and elderly and rural people are easily affected (Zhang et al. 2022). Because of severe itching and recurrent lesions, CU has significant impact on patients’ quality of life, including sleep disturbance, anxiety, depression, and social dysfunction. Moreover, the high consumption of medical resources and other indirect costs for the treatment of CU causes a major public health challenge (Gonçalo et al. 2021).

Although the pathogenesis of CU has not been clearly interpreted yet, mast cells (MCs) and histamine in skin play an important role (He et al. 2021). Therefore, H1-antihistamines (H1-AHs) are recommended as the first-line treatment in the guideline for the management of CU (Zuberbier et al. 2022). However, some patients with CU still do not achieve the desired effect even at 4-fold standard dose of H1-AHs or the combination of different H1-AHs (Pereyra-Rodriguez et al. 2020). Although omalizumab and cyclosporin could be used in the patients who are unresponsive to H1-AHs (He et al. 2021), the high cost of omalizumab and adverse effects of cyclosporin, including nephrotoxicity and elevated blood pressure, may limit their long-term use for the treatment of CU (Matsubara et al. 2021; Zuberbier et al. 2022).

In China, *Tripterygium* glycosides (TG) is a traditional Chinese medicine (TCM) extracted and purified from the roots of *Tripterygium wilfordii* Hook. F (Celastraceae) (TwHF). Because of the anti-inflammatory and immunosuppressive effects, TG has been applied to treat some autoimmune and inflammatory diseases in China, including rheumatoid arthritis, Sjögren’s syndrome, and diabetic nephropathy (Guo et al. 2021; Luo et al. 2021; Geng et al. 2022). In recent years, some clinical trials have indicated that TG as an add-on treatment could improve the efficacy in comparison with H1-AH alone in adults with CU (Wei et al. 2010; Xing and Feng 2017), and TG is recommended as the second-line treatment in the Chinese guideline for urticaria (CSD, CUR 2019).

Although two recent meta-analyses regarding TG for CU have been published (Liu et al. 2018; Shi et al. 2019), there are some limitations in them. They only consisted of the articles until July 2018, and did not explicate the definitions of efficacy outcomes. Two studies did not assess the effects of TG with different treatment durations and different dosages, and the type of H1-AH was restricted to desloratadine in the one study (Shi et al. 2019). Therefore, in order to provide thorough evidence for clinical practice, this meta-analysis systematically re-evaluates and updates the efficacy and safety of TG as an add-on treatment in adults with CU with regard to the latest published articles.

## Materials and methods

This meta-analysis was conducted based on the Preferred Reporting Items for Systematic Reviews and Meta-Analyses (PRISMA) Statement (Page et al. 2021), and it has been registered in the PROSPERO (ID number: CRD42022322869).

### Search strategy

Four Chinese literature databases and four English literature databases were searched from their inceptions to May 31, 2022, including China National Knowledge Infrastructure (CNKI), WangFang, Chinese Scientific Journal Database (VIP), Chinese Biological Medicine Database (SinoMed), PubMed, Cochrane Library, Embase, and Web of Science. Medical subject heading (MeSH), title, and abstract were combined to retrieve the relevant studies. The following search strategy was used: {(urticaria) [Mesh] OR (urticaria) [Title/Abstract]} AND {(*Tripterygium*) [Title/Abstract] OR (Lei Gong Teng) [Title/Abstract] OR (Leigongteng) [Title/Abstract]}. The references of the included studies were also manually searched to screen for additional qualified studies. The literature search was conducted by two independent authors (M Li and Y Li), and any disagreements were resolved by the third author (LJ Xiang).

### Eligibility criteria

#### Inclusion criteria

Studies meeting the following criteria were included: (1) Only randomized controlled trials (RCTs) published in Chinese or English were included. (2) Adults were diagnosed with CU defined as the occurrence of wheals and/or angioedema for 6 weeks or more, regardless of gender, disease course, and disease severity. (3) The test groups were treated with TG combined with H1-AH, and the control groups were treated with H1-AH alone. There were no restrictions on the dosage of each drug and treatment duration. (4) The primary outcomes included cure rate and total efficacy rate. Cure rate was defined as the proportion of patients with no wheal and itch after the treatment, and total efficacy rate was defined as the percentage of patients achieving at least 60% improvement in disease severity from baseline after the treatment. The secondary outcomes included score of pruritus, score of wheal number, score of wheal size, the level of immunoglobulin E (IgE) in serum and adverse events (AEs). The 4-grade scoring system was used to assess the degrees of pruritus, wheal number, and wheal size. No itch and wheals were scored 0. Mild itch, 6 wheals or less/24 h, or the maximum diameter of wheals <5 mm was marked as one point. Moderate itch, 7 to 12 wheals/24 h, or the maximum diameter of wheals from 5 to 20 mm was evaluated as two points. Three points meant severe itch, 13 wheals or more/24 h, or the maximum diameter of wheals >20 mm. In terms of AEs, different symptoms and laboratory tests during the treatments were recorded.

#### Exclusion criteria

Exclusion criteria were as follows: reviews, case reports, abstracts, cell experiments, animal experiments, non-RCTs, pregnant or lactational women, duplicated publications, and studies with unavailable data.

### Literature screening and data extraction

Based on the eligibility criteria, two independent authors (M Li and Y Li) screened the title and abstract of each study and excluded irrelevant studies. Then the full texts of the remaining studies were obtained to confirm the included studies. The third author (LJ Xiang) dealt with the discrepancy between two authors.

Two authors (M Li and Y Li) independently extracted the following data from each included trial *via* a structured data collection table: study characteristics (the first author’s name, publication year, country, and the number of research center), patients demographics (type of CU, sample size, gender, age, and disease course), interventions and comparisons (type of H1-AH, dosages of TG and H1-AH, and treatment duration) and outcomes. In case of any disagreement, the third author (LJ Xiang) was consulted.

### Assessment of methodological quality

By using the Cochrane collaboration’s tool (Higgins et al. 2011), two authors (M Li and Y Li) evaluated the methodological quality of each included trial in an independent way. The included seven items were as follow: random sequence generation (selection bias), allocation concealment (selection bias), blinding of participants and personnel (performance bias), blinding of the outcome assessment (detection bias), incomplete outcome data (attrition bias), selective reporting (reporting bias), and other bias. The baselines of disease severity between two groups were considered as the source of other bias. Each item was classified as low, high, or unclear risk of bias. The disagreement between two authors was resolved by discussing with the third author (LJ Xiang).

### Statistical analysis

Review Manager (RevMan) 5.3 and Stata 12.0 were used for data analysis. Risk ratio (RR) with 95% confidence interval (CI) was calculated for dichotomous data, whereas mean difference (MD) or standardized mean difference (SMD) with 95% CI was calculated for continuous data. The heterogeneity among studies was assessed by using the Chi-squared test and *I*^2^ statistic. If *I*^2^ >50% or *p* < 0.1, indicating a significant heterogeneity, subgroup analysis could be conducted based on treatment durations, dose regimens of TG, or types of H1-AHs to detect the origin of heterogeneity, and a random-effect model was used if the heterogeneity could not be analyzed. Otherwise, a fixed-effect model was applied for statistical analysis. The stabilities of the pooled results were evaluated by using the leave-one-out method to test the impact of each study on the pooled results. When 10 or more studies were included in the same outcome, the potential publication bias was assessed by using funnel plot and Egger test. A two tailed *p* < 0.05 was considered to be statistically significant.

## Results

### Selection of the included studies

**Figure 1.**
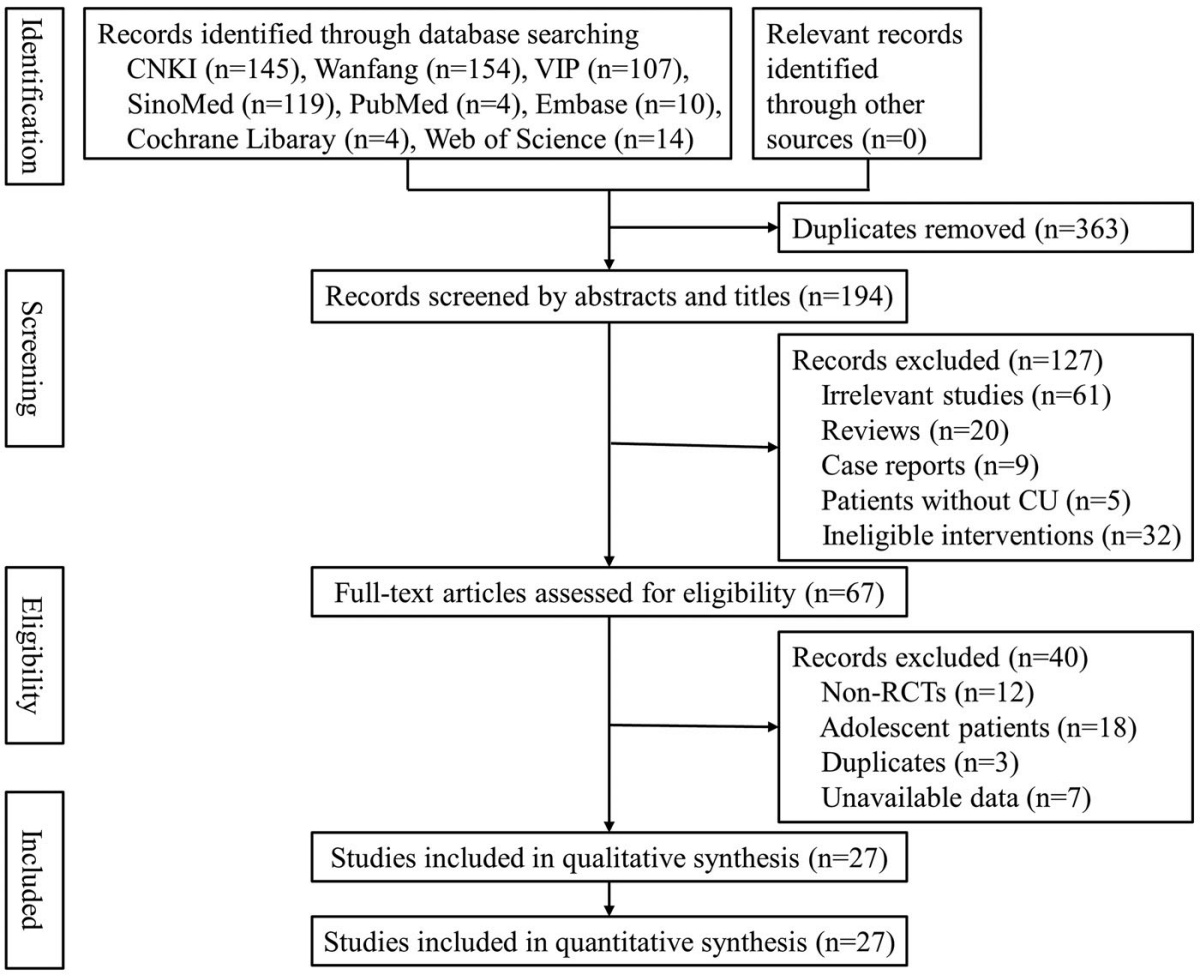
The flow diagram of study selection.

A total of 557 studies were retrieved from eight literature databases. After removing 363 duplicates, 194 studies remained for further selection. Among them, 127 studies were removed due to ineligible titles and abstracts, and 40 studies were excluded after screening the full texts. Finally, 27 studies (Pi et al. 2006; He et al. 2009; Wang 2010; Qian and Zhang 2011; Yang 2011; Xie et al. 2012; He 2013; Wang 2014; Zhou 2014; Chen 2016; Liu 2016; Chen 2017; Chen et al. 2017; Deng et al. 2017; Pei 2017; Qin and Han 2017; Wang 2018; Xu 2018; Zhao 2018; Zhou et al. 2018; Zhu 2018; Xiao and Zhang 2019; Yang 2019; Gao 2020; Long et al. 2021; Zhang 2021; Zhang and Ma 2021) were included in this meta-analysis (Figure 1).

### Characteristics of the included studies

All 27 included studies were single-center trials conducted in China. The publication years of them ranged from 2006 to 2021. Among them, 16 studies (Pi et al. 2006; He et al. 2009; Yang 2011; Xie et al. 2012; He 2013; Wang 2014; Zhou 2014; Liu 2016; Chen 2017; Chen et al. 2017; Deng et al. 2017; Pei 2017; Zhao 2018; Zhou et al. 2018; Zhu 2018; Long et al. 2021) recruited patients with CSU, while the remaining 11 studies had no limitation on the types of CU. A total of 2788 patients were enrolled, and half of them were treated with TG combined with H1-AH or H1-AH alone, respectively. The maximum and minimum doses of TG in all included studies were 60 mg/d and 10 mg/d, respectively, and 7 kinds of H1-AHs were applied in two groups, including mizolastine, ebastine, loratadine, cetirizine, desloratatine, fexofenadine, and setastine. The treatment durations in all studies ranged from 4 to 12 weeks. The basic characteristics of the included studies are shown in Table 1.

**Table 1.** The basic characteristics of the included studies.

Study ID	Types of CU	Sample size (Male/Female)		Age (years)		Interventions		Treatment duration (weeks)	Outcomes
		T	C	T	C	T	C		
Pi et al. (2006)	CSU	21	22	18-55	18-55	TG 20 mg bid + Mizolastine 10 mg qd	Mizolastine 10 mg qd	12	①②⑦
He et al. (2009)	CSU	25	25	≥18	≥18	TG 20 mg tid + Mizolastine 10 mg qd	Mizolastine 10 mg qd	4	②⑦
Wang (2010)	CU	41 (18/23)	41 (19/22)	20-62	19-63	TG 30 mg qd + Ebastine 10 mg qd	Ebastine 10 mg qd	4	⑥⑦
Qian and Zhang (2011)	CU	T1:62 (33/29) T2:61 (28/33) T3:57 (30/27)	C1:61 (33/28) C2:60 (34/26) C3:59 (34/25)	18-65	18-65	T1: TG 20 mg tid + Loratadine 10 mg qd T2: TG 20 mg tid + Mizolastine 10 mg qd T3: TG 20 mg tid + Cetirizine 10 mg qd	C1: Loratadine 10 mg qd C2: Mizolastine 10 mg qd C3: Cetirizine 10 mg qd	4	⑦
Yang (2011)	CSU	48	48	31-64	31-64	TG 20 mg tid + Desloratadine 5 mg qd	Desloratadine 5 mg qd	4	②
Xie et al. (2012)	CSU	100 (44/56)	100 (41/59)	35-61	32-58	TG 10 mg tid + Desloratadine 5 mg qd	Desloratadine 5 mg qd	4	②
He (2013)	CSU	105	105	30-65	30-65	TG 20 mg tid + Desloratadine 5 mg qd	Desloratadine 5 mg qd	4	②⑥⑦
Zhou (2014)	CSU	60	60	31-63	31-63	TG 10 mg tid + Desloratadine 5 mg qd	Desloratadine 5 mg qd	4	①⑦
Wang (2014)	CSU	32	32	19-63	19-63	TG 10 mg qd + Desloratadine 5 mg qd	Desloratadine 5 mg qd	4	①⑦
Chen (2016)	CU	52	52	18-68	18-68	TG 10 mg qd + Ebastine 10 mg qd	Ebastine 10 mg qd	4	⑦
Liu (2016)	CSU	60 (32/28)	60 (35/25)	25-71	18-67	TG 20 mg tid + Desloratadine 5 mg qd	Desloratadine 5 mg qd	4	⑦
Chen (2017)	CSU	45 (21/24)	45 (23/22)	25-71	24-72	TG 10 mg tid + Desloratadine 5 mg qd	Desloratadine 5 mg qd	4	①⑦
Deng et al. (2017)	CSU	60	60	37-59	37-59	TG 10 mg tid + Desloratadine 5 mg qd	Desloratadine 5 mg qd	4	①
Chen et al. (2017)	CSU	42 (27/15)	42 (29/13)	35-48	35-48	TG 10 mg qd + Desloratadine 5 mg qd	Desloratadine 5 mg qd	4	②⑦
Pei (2017)	CSU	25	25	18-65	18-65	TG 20 mg tid + Fexofenadine 60 mg bid	Fexofenadine 60 mg bid	4	②⑦
Qin and Han (2017)	CU	35 (19/16)	35 (20/15)	18-56	18-53	TG 10 mg tid + Ebastine 10 mg qd	Ebastine 10 mg qd	4	②⑦
Xu (2018)	CU	55 (28/27)	55 (30/25)	23-64	21-62	TG 20 mg tid + Ebastine 10 mg qd	Ebastine 10 mg qd	4	⑦
Zhou et al. (2018)	CSU	59 (37/22)	59 (39/20)	20-61	21-60	TG 20 mg tid + Desloratadine 5 mg qd	Desloratadine 5 mg qd	8	②③
Zhao (2018)	CSU	38 (18/20)	38 (19/19)	20-70	21-71	TG 10 mg tid + Desloratadine 2.5 mg qd	Desloratadine 2.5 mg qd	4	①
Zhu (2018)	CSU	35 (25/10)	35 (24/11)	21-63	22-64	TG 10 mg tid + Desloratadine 5 mg qd	Desloratadine 5 mg qd	4	①⑤⑦
Wang (2018)	CU	36 (21/15)	36 (19/17)	19-63	21-60	TG 10 mg qd + Ebastine 10 mg qd	Ebastine 10 mg qd	4	⑦
Yang (2019)	CU	24 (13/11)	24 (14/10)	19-63	20-61	TG 20 mg tid + Desloratadine 5 mg qd	Desloratadine 5 mg qd	4	②
Xiao and Zhang (2019)	CU	40 (22/18)	40 (19/21)	18-75	18-75	TG 20 mg tid + Setastine 1 mg bid	Setastine 1 mg bid	12	②⑦
Gao (2020)	CU	45 (28/17)	45 (27/18)	19-65	18-64	TG 10 mg tid + Ebastine 10 mg qd	Ebastine 10 mg qd	4	②⑦
Zhang and Ma (2021)	CU	28 (16/12)	28 (17/11)	18-65	18-64	TG 10 mg tid + Ebastine 10 mg qd	Ebastine 10 mg qd	4	③④⑤⑦
Zhang (2021)	CU	63 (34/29)	62 (35/27)	21-64	21-61	TG 10 mg tid + Mizolastine 10 mg qd	Mizolastine 10 mg qd	4	②③④⑤⑥
Long et al. (2021)	CSU	40 (23/17)	40 (24/16)	32-57	33-56	TG 20 mg tid + Desloratadine 5 mg qd	Desloratadine 5 mg qd	8	③④⑦

T: test group; C: control group; TG: Tripterygium glycosides; CU: chronic urticaria; CSU: chronic spontaneous urticaria; qd: once per day; bid: twice per day; tid: three times per day.

① cure rate; ② total efficacy rate; ③ pruritus score; ④ the number of wheals; ⑤ the size of wheals; ⑥ the levels of IgE in serum; ⑦ adverse events.

### Quality assessment of the included studies

**Figure 2.**
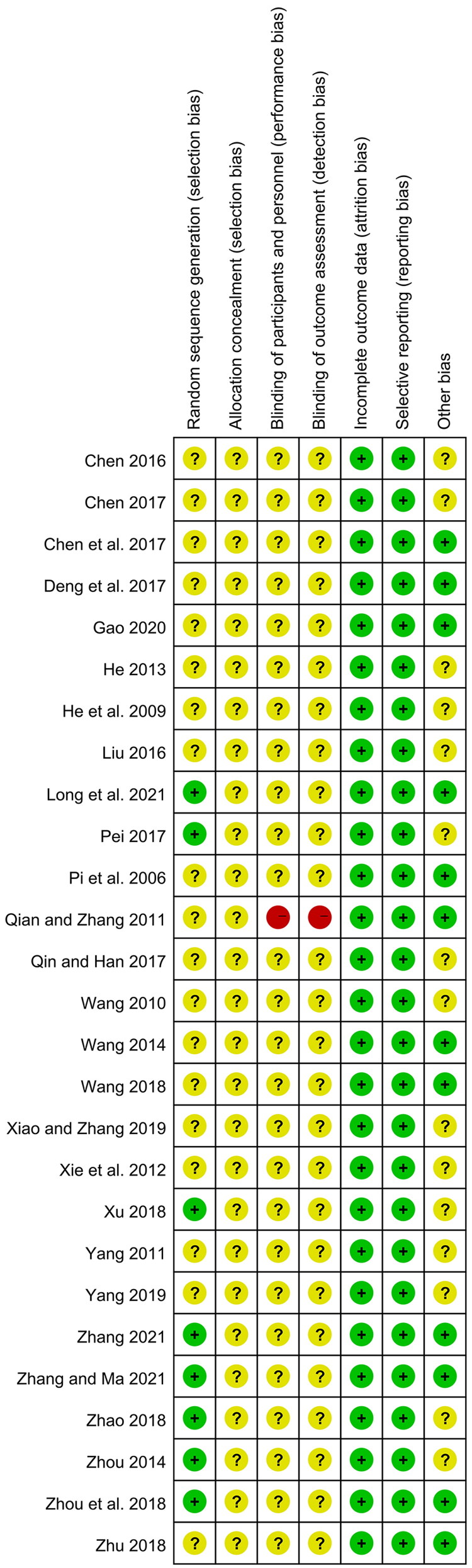
Risk of bias summary of the included studies.

Although all included studies mentioned randomization, only 8 studies described the detailed randomization methods and were evaluated as low risk, including 6 (Zhou 2014; Pei 2017; Zhao 2018; Zhou et al. 2018; Long et al. 2021; Zhang 2021) with a random number table, 1 (Zhang and Ma 2021) with a computer random number generator, and 1 (Xu 2018) with drawing of lots. All studies did not mention the allocation concealment and were rated as unclear risk. In terms of performance bias and detection bias, except for one open-label trial (Qian and Zhang 2011) marked as high risk, the remaining 26 studies were judged as unclear risk due to the insufficient information. Because all studies provided complete data and reported all outcomes. they were rated as low risks of detection bias and attrition bias. With regard to other bias, the baselines of disease severity between two groups were comparable in 12 studies (Pi et al. 2006; Qian and Zhang 2011; Wang 2014; Chen et al. 2017; Deng et al. 2017; Wang 2018; Zhou et al. 2018; Zhu 2018; Gao 2020; Long et al. 2021; Zhang 2021; Zhang and Ma 2021), and they were assessed as low risk, while the remaining 15 studies did not report the relevant data and were judged as unclear risk (Figure 2).

### Primary outcomes

#### Cure rate

**Figure 3.**
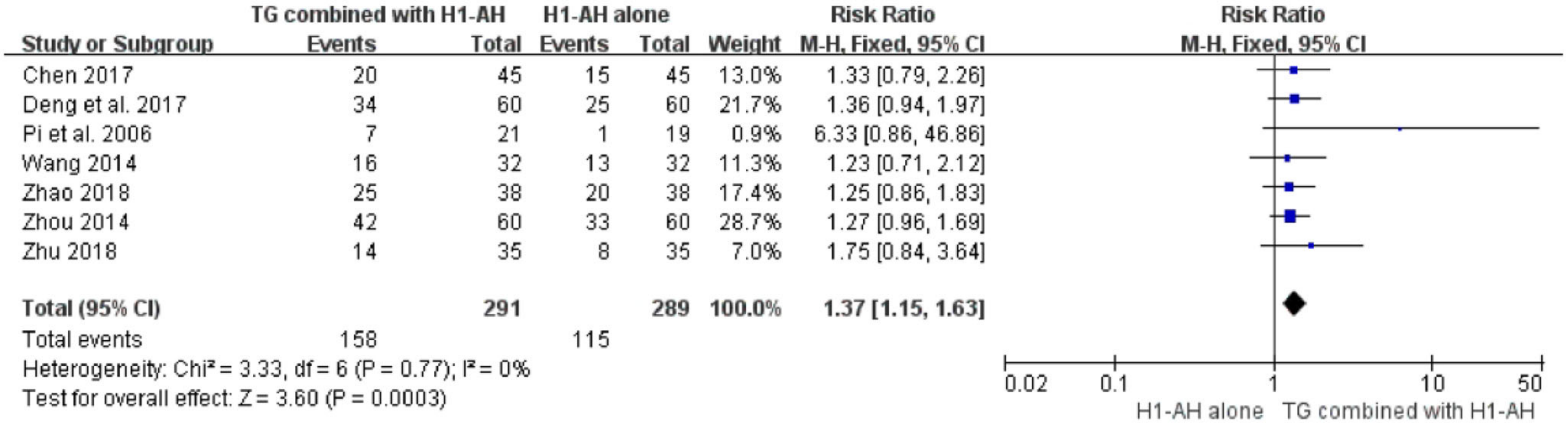
Forest plot for cure rates between TG plus H1-AH and H1-AH alone.

Seven studies (Pi et al. 2006; Wang 2014; Zhou 2014; Chen 2017; Deng et al. 2017; Zhao 2018; Zhu 2018) reported the cure rates between TG plus H1-AH and H1-AH alone, and 580 patients were included. Due to no significant heterogeneity among the studies (*p* = 0.77, *I*^2^ = 0%), a fixed-effect model was used. The pooled results showed that TG combined with H1-AH significantly improved cure rate in comparison with H1-AH alone (RR = 1.37; 95% CI = 1.15 to 1.63, *p* = 0.0003) (Figure 3).

#### Total efficacy rate

**Figure 4.**
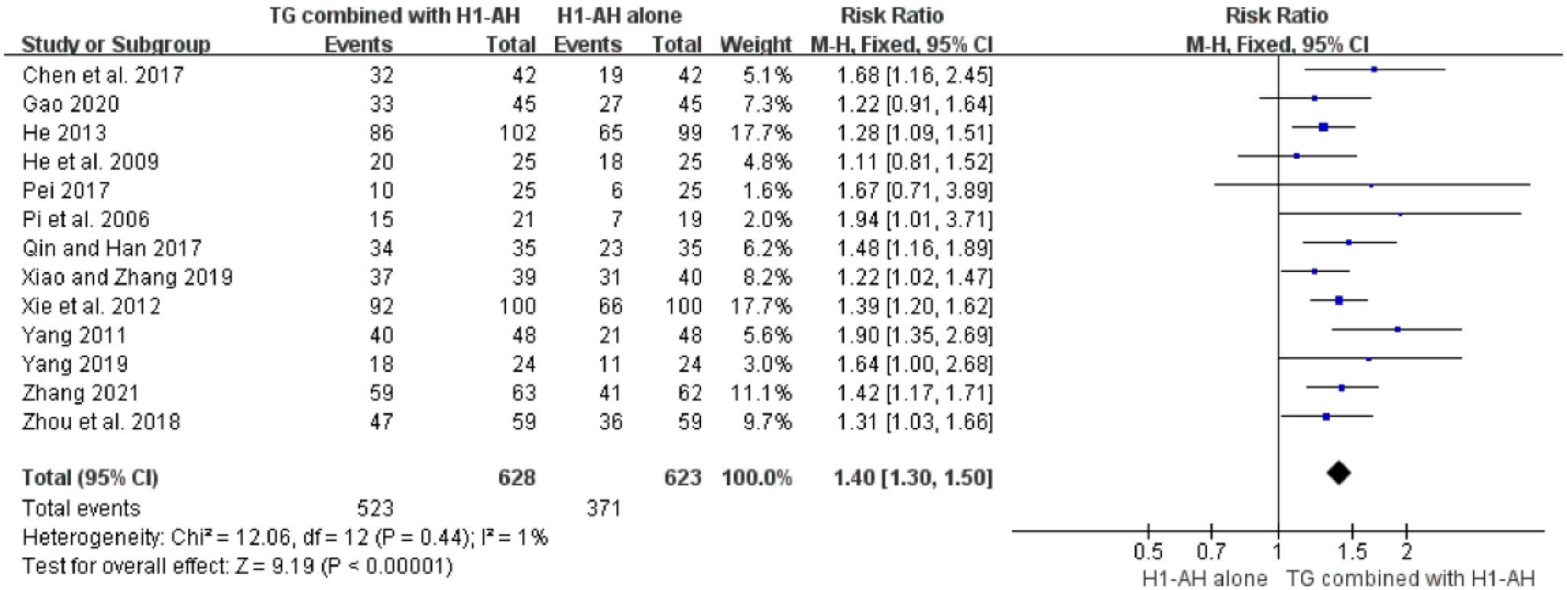
Forest plot for total efficacy rates between TG plus H1-AH and H1-AH alone.

The total efficacy rates between TG plus H1-AH and H1-AH alone were evaluated in 13 studies (Pi et al. 2006; He et al. 2009; Yang 2011; Xie et al. 2012; He 2013; Chen et al. 2017; Pei 2017; Qin and Han 2017; Zhou et al. 2018; Xiao and Zhang 2019; Yang 2019; Gao 2020; Zhang 2021), and 1251 patients were involved. Because the heterogeneity among the studies was not significant (*p* = 0.44, *I*^2^ = 1%), a fixed-effect model was performed. The pooled results demonstrated that total efficacy rates of the TG combined with H1-AH groups were significantly higher than those of the H1-AH alone groups (RR = 1.40, 95% CI = 1.30 to 1.50, *p* < 0.00001) (Figure 4).

**Figure 5.**
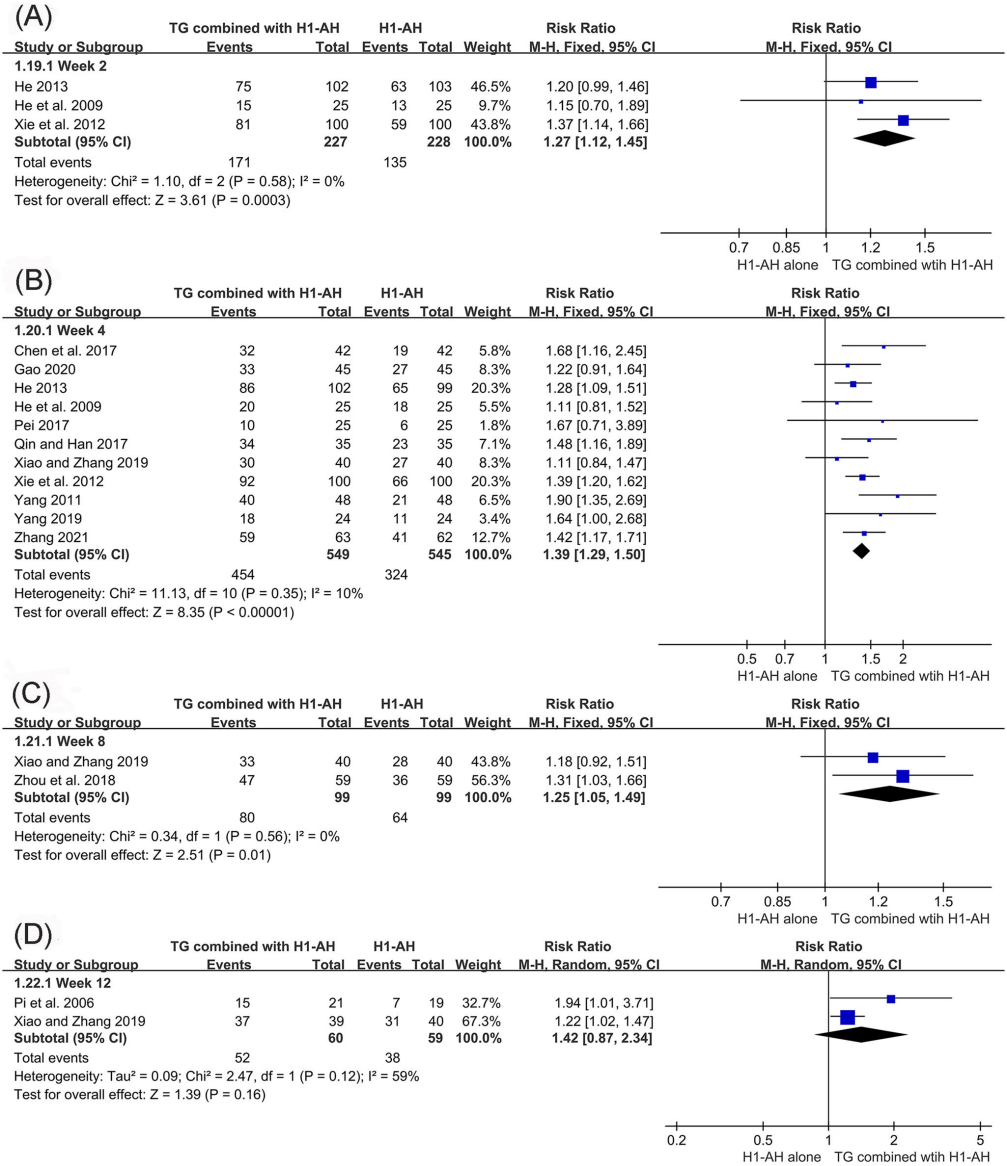
Forest plots for total efficacy rates after different treatment durations. (A) week 2, (B) week 4, (C) week 8, (D) week 12.

**Figure 6.**
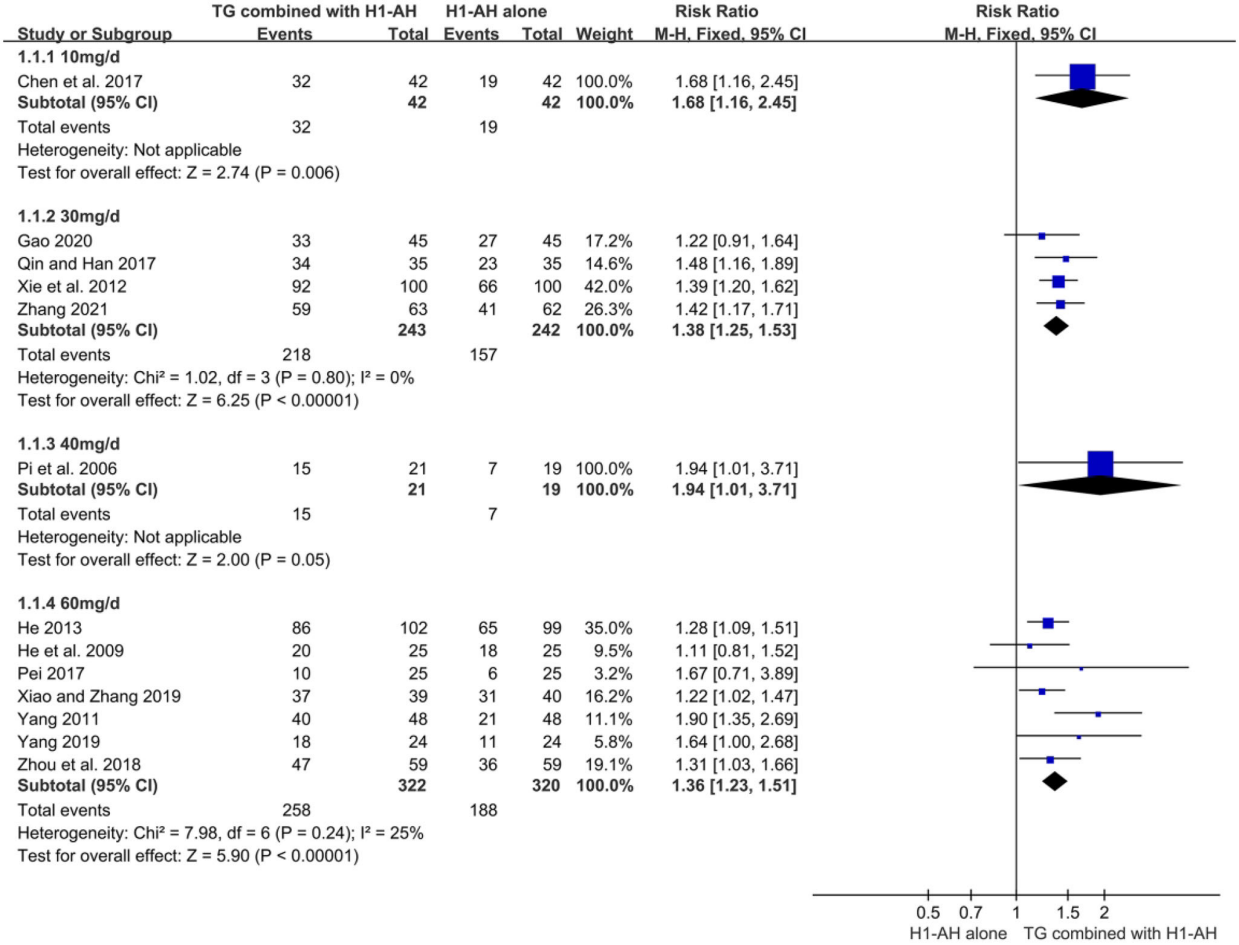
Forest plots for total efficacy rates with different dose regimens of TG.

In addition, subgroup analyses were conducted based on different treatment durations and dose regimens of TG. The pooled results revealed that the rates of the combination therapy were significantly higher than those of monotherapy after 2 weeks of treatment (RR = 1.27, 95% CI = 1.12 to 1.45, *p* = 0.0003), and the advantage could be maintained at week 4 (RR = 1.39, 95% CI = 1.29 to 1.50, *p* < 0.00001) and week 8 (RR = 1.25, 95% CI = 1.05 to 1.49, *p* = 0.01). Although the combination therapy had a higher rate than monotherapy at week 12, the differences between two groups were not statistically significant (RR = 1.42, 95% CI = 0.87 to 2.34, *p* = 0.16) (Figure 5). Moreover, the pooled results also showed that TG as an add-on treatment could significantly improve the total efficacy rate, regardless of the doses of 10 mg/d (RR = 1.68, 95% CI = 1.16 to 2.45, *p* = 0.006), 30 mg/d (RR = 1.38, 95% CI = 1.25 to 1.53, *p* < 0.00001), 40 mg/d (RR = 1.94, 95% CI = 1.01 to 3.71, *p* = 0.05), and 60 mg/d (RR = 1.36, 95% CI = 1.23 to 1.51, *p* < 0.00001) (Figure 6).

### Secondary outcomes

#### Score of pruritus

**Figure 7.**
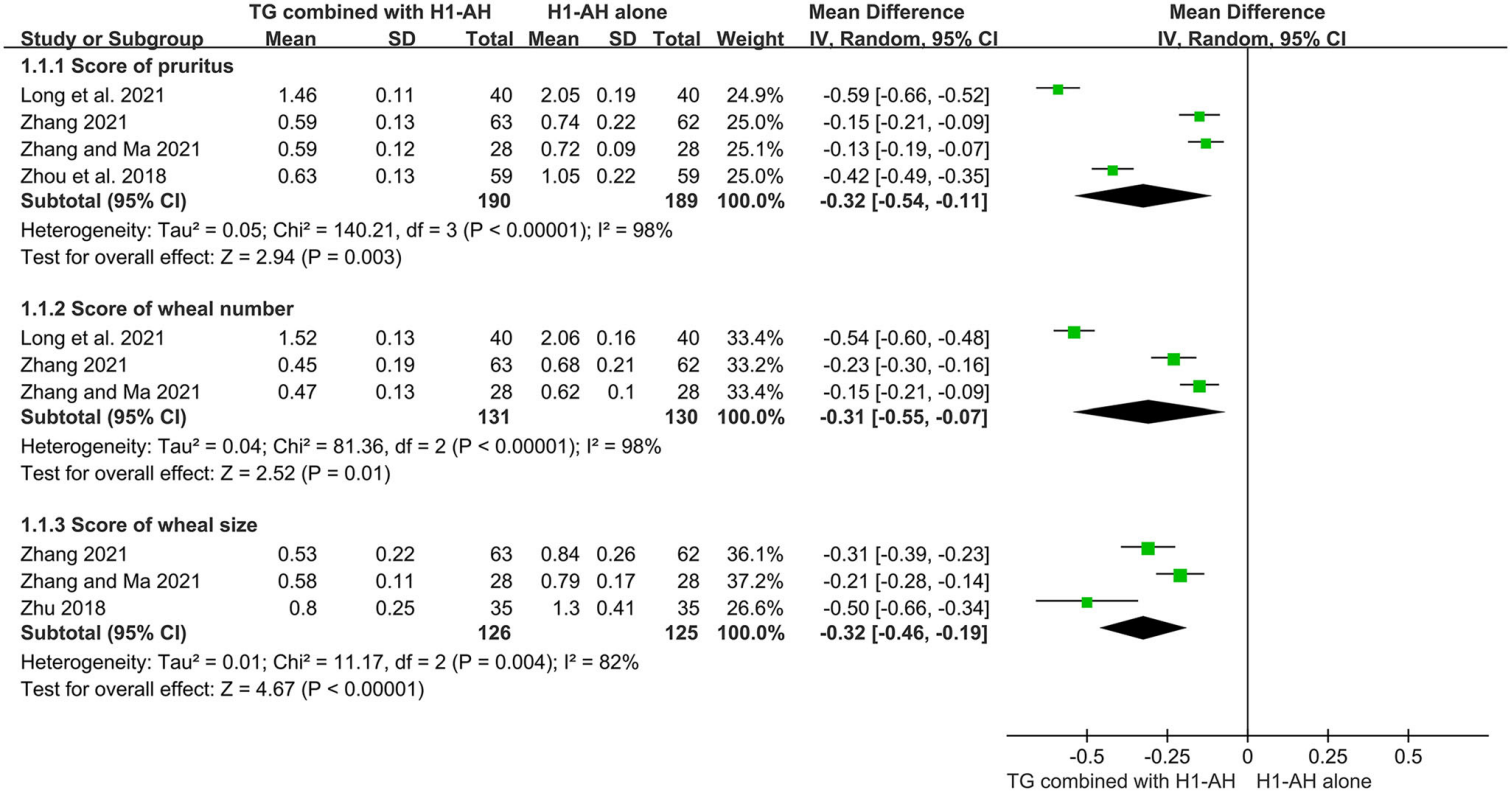
Forest plots for scores of pruritus, wheal number, and wheal size between TG plus H1-AH and H1-AH alone.

Four studies (Zhou et al. 2018; Long et al. 2021; Zhang 2021; Zhang and Ma 2021) evaluated scores of pruritus between two groups, and 379 patients were included. Due to a significant heterogeneity among the studies (*p* < 0.00001, *I*^2^ = 98%), which might be attributed to different baselines or treatment durations. a random-effect model was used. The pooled results displayed that TG plus H1-AH could better alleviate itch than H1-AH alone (MD = −0.32, 95% CI = −0.54 to −0.11, *p* = 0.003) (Figure 7).

#### Score of wheal number

The numbers of wheals between two groups were calculated in three studies (Long et al. 2021; Zhang 2021; Zhang and Ma 2021), which recruited 261 patients. The heterogeneity among the studies (*p* < 0.00001, *I*^2^ = 98%) was significant, and different baselines or treatment durations might result in this phenomenon, therefore a random-effect model was applied. The pooled results showed that the numbers of wheals in the TG plus H1-AH groups were significantly lower than those in the H1-AH alone groups (MD = −0.31, 95% CI = −0.55 to −0.07, *p* = 0.01) (Figure 7).

#### Score of wheal size

Three studies (Zhu 2018; Zhang 2021; Zhang and Ma 2021) measured the sizes of wheals between two groups, and 251 patients were included. Because of a significant heterogeneity among the studies (*p* = 0.004, *I*^2^ = 82%), which might result from different baselines or types of H1-AHs, a random-effect model was used. The pooled results revealed that the combination therapy significantly decreased the sizes of wheals in comparison with monotherapy (MD = −0.32, 95% CI = −0.46 to −0.19, *p* < 0.00001) (Figure 7).

#### The level of IgE in serum

**Figure 8.**
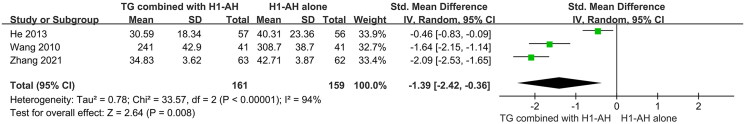
Forest plot for the levels of IgE in serum between TG plus H1-AH and H1-AH alone.

The levels of IgE in serum were monitored in three studies (Wang 2010; He 2013; Zhang 2021), which included 320 patients. Due to different units of test methods, SMD was used. Moreover, the heterogeneity among the studies was significant (*p* < 0.00001, *I*^2^ = 94%), which might be related to different baselines, therefore a random-effect model was conducted. The pooled results revealed that TG plus H1-AH significantly reduced the levels of IgE in serum than H1-AH alone (SMD = −1.39, 95% CI = −2.42 to −0.36, *p* = 0.008) (Figure 8).

#### Adverse events

**Figure 9.**
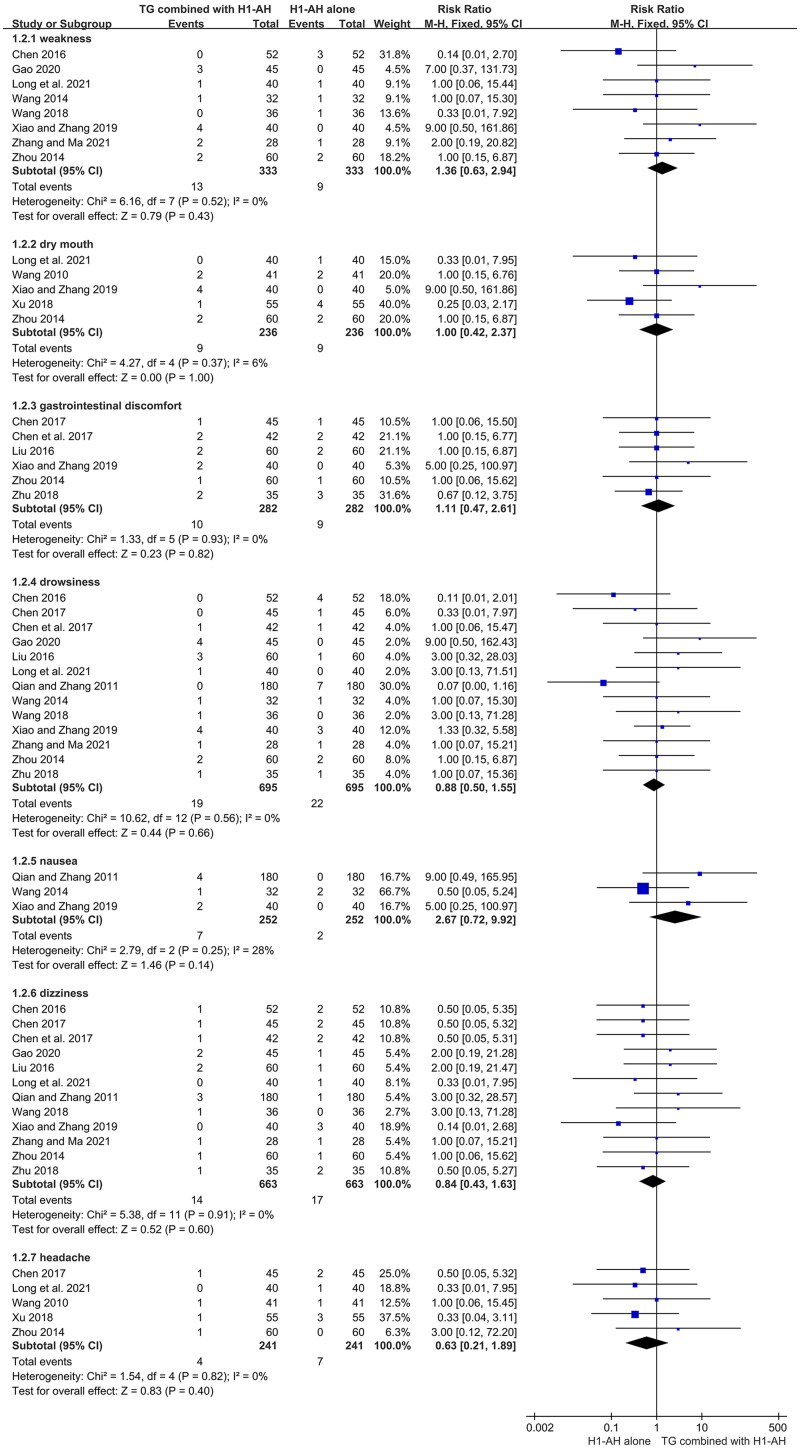
Forest plots for the incidences of weakness, dry mouth, gastrointestinal discomfort, drowsiness, nausea, dizziness, and headache.

A total of 20 studies (Pi et al. 2006; He et al. 2009; Wang 2010; Qian and Zhang 2011; He 2013; Wang 2014; Zhou 2014; Chen 2016; Liu 2016; Chen 2017; Chen et al. 2017; Pei 2017; Qin and Han 2017; Wang 2018; Xu 2018; Zhu 2018; Xiao and Zhang 2019; Gao 2020; Long et al. 2021; Zhang and Ma 2021) evaluated the AEs between two groups during the treatments, and 2005 patients were included. A few patients in two groups suffered from AEs, most of which were mild and tolerable. The common AEs included weakness, dry mouth, gastrointestinal discomfort, drowsiness, nausea, dizziness, and headache. The pooled results showed that the incidences between two groups were not significantly different in terms of weakness (RR = 1.36, 95% CI = 0.63 to 2.94, *p* = 0.43, *I*^2^ = 0%), dry mouth (RR = 1.00, 95% CI = 0.42 to 2.37, *p* = 1.00, *I*^2^ = 6%), gastrointestinal discomfort (RR = 1.11, 95% CI = 0.47 to 2.61, *p* = 0.82, *I*^2^ = 0%), drowsiness (RR = 0.88, 95% CI = 0.50 to 1.55, *p* = 0.66, *I*^2^=0%), nausea (RR = 2.67, 95% CI = 0.72 to 9.92, *p* = 0.14, *I*^2^ = 28%), dizziness (RR = 0.84, 95% CI = 0.43 to 1.63, *p* = 0.60, *I*^2^ = 0%), and headache (RR = 0.63, 95% CI = 0.21 to 1.89, *p* = 0.40, *I*^2^ = 0%) (Figure 9). In addition, two female patients receiving TG plus H1-AH suffered from amenorrhea, while those receiving H1-AH alone did not.

**Figure 10.**
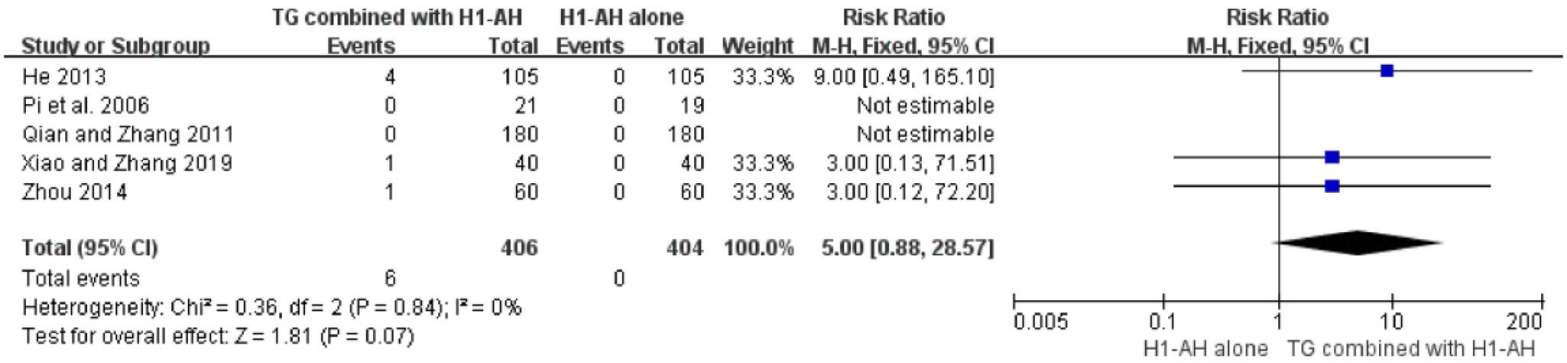
Forest plot for the incidences of elevated liver enzyme levels between TG plus H1-AH and H1-AH alone.

Laboratory tests were also conducted in 6 studies (Pi et al. 2006; He et al. 2009; Qian and Zhang 2011; He 2013; Zhou 2014; Xiao and Zhang 2019), including blood routine, urine routine, and liver and renal functions. Six patients with a slightly elevated levels of liver enzymes (alanine transaminase (ALT) or aspartate transaminase (AST)) were observed in the TG plus H1-AH groups, while no similar case occurred in the H1-AH alone groups. The pooled results showed that the incidences between two groups were not significantly different (RR = 5.00, 95% CI = 0.88 to 28.57, *p* = 0.07) (Figure 10). No abnormal changes of blood routine, urine routine, and renal function in two groups were reported.

### Sensitivity analysis

Sensitivity analysis was conducted by omitting one study as a time and calculating the pooled results for the remaining studies. The results revealed that no significant change of the pooled results was observed after excluding any single study. Therefore, the pooled results of this meta-analysis were relatively robust.

### Publication bias

**Figure 11.**
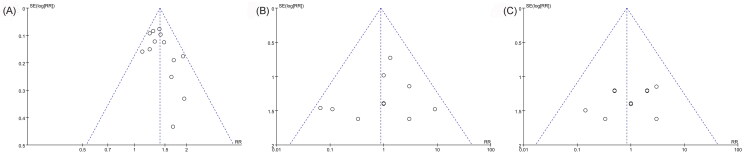
Funnel plots of total efficacy rate (A), the incidence of drowsiness (B), and the incidence of dizziness (C).

**Figure 12.**
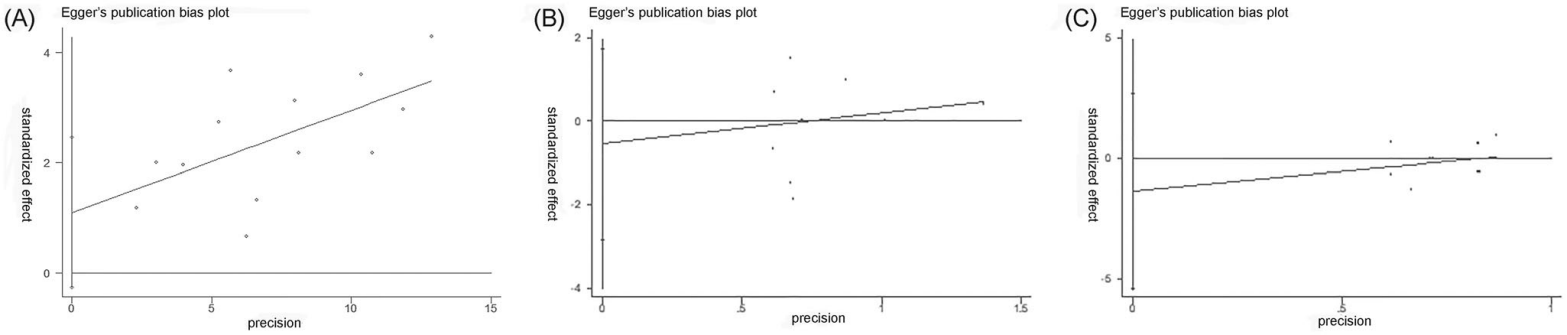
Egger tests of total efficacy rate (A), the incidence of drowsiness (B), and the incidence of dizziness (C).

Because 10 or more studies were included in the following three outcomes: total efficacy rate and the incidences of drowsiness and dizziness, the publication biases on them were evaluated by funnel plot and Egger test. All nearly symmetrical funnel plots of three outcomes indicated relatively low possibilities of publication bias (Figure 11), and the negative results of Egger tests also demonstrated that there were no significant publication biases on total efficacy rate (*p* = 0.103), the incidence of drowsiness (*p* = 0.607), and the incidence of dizziness (*p* = 0.470) (Figure 12).

## Discussion

CU is a recurrent inflammatory skin disease with a large population of patients, and it has serious impact on patients’ life quality and healthcare system. For the management of CU, H1-AH is recommended as the first-line treatment to relieve symptom. However, due to the complicated mechanisms of CU, the effect of H1-AH has not been satisfactory with patients with refractory CU. In China, TCM plays an important role in the treatment of CU and may be a supplement or replacement for western medicine. TG has been widely applied as a standard therapy in CU for decades. However, there is no comprehensive meta-analysis regarding TG for the treatment of CU. Therefore, this meta-analysis collected 27 RCTs and showed that TG as an add-on treatment had good effects on relieving symptoms of CU, and the AEs of TG were mild and tolerable.

Currently, the pathogenic mechanisms of CU remain unclear. Skin MCs are considered as the primary effector cells, and a variety of mediators produced and secreted from them could lead to vasodilation, fluid exudation and increased vascular permeability, such as histamine, leukotrienes, and prostaglandins. (Altman and Chang 2013). The mechanisms of MC activation involve autoimmunity and non-autoimmunity. On the one hand, IgE autoantibodies and IgG autoantibodies could be detected in some patients with CU, which could activate IgE receptors on MCs and lead to degranulation and a subsequent biochemical response. On the other hand, some non-autoimmune mechanisms participate in the pathogenesis of other CU patients, including coagulation cascade, infection, and stress (He et al. 2021). The plasma levels of some coagulation biomarkers were increased in CU patients, such as activated factor VII, D-dimer, and fibrin degradation products (Asero et al. 2007; Takahagi et al. 2010). In addition, eosinophils and different T cell subsets also play important roles. Eosinophils could not only promote the recruitment, maturation and degranulation of MCs in the tissues by generating eosinophil peroxidase and eosinophil cationic protein, but also contribute to the persistence of wheals by releasing leukotrienes and prostaglandins (Altrichter et al. 2020). Some studies displayed that the proportions of Th2 cells and Th17 cells in lesions were significantly higher in patients with CU, and the plasma levels of Th2-related and Th17-related cytokines were also elevated, such as interleukin (IL)-5, IL-6, IL-13, IL-21, and IL-23 (Moy et al. 2016; Chen et al. 2018). In terms of the ingredients, TG includes diterpenoids (e.g., triptolide), triterpenoids (e.g., celastrol, wilforlide A, wilforlide B), and alkaloids (e.g., wilforine, wilfortrine) (Ru et al. 2019). Our meta-analysis showed that TG as an add-on treatment could significantly decrease the levels of serum IgE, and other studies also revealed that the levels of rheumatoid factor, an autoantibody against the fragments of IgG, in rheumatoid arthritis patients and Sjögren’s syndrome patients were significantly reduced after the treatment of TG (Lu et al. 2021; Gan et al. 2022). These results demonstrated that TG could alleviate autoimmune reactions. On the other hand, two trials (Zhong et al. 2020; Ren et al. 2022) revealed that TG as adjuvant therapy significantly reduced the levels of D-dimer and fibrinogen in Henoch-Schonlein purpura nephritis patients and ankylosing spondylitis patients, indicating the effect of TG on regulating coagulation system. Furthermore, some vitro studies also showed that celastrol could inhibit the proliferation of MCs and reduce the release of histamine (Yao et al. 2017; Zhang et al. 2018). In addition, some vitro studies displayed that triptolide could not only promote the apoptosis of eosinophils and T cells, but also decrease the levels of IL-2, IL-4, IL-5, IL-13, IL-17 and IL-23 (Lin et al. 2000; 2001; Li et al. 2002; Dai et al. 2013; Yang et al. 2015). Therefore, TG could relieve the symptoms of CU through different mechanisms.

With regard to the efficacy of TG, this meta-analysis showed that TG combined with H1-AH could significantly alleviate itch and reduce the number and size of wheals in comparison with H1-AH alone. Meanwhile, TG plus H1-AH was superior to H1-AH alone in cure rate and total efficacy rate. These data demonstrated that TG as an add-on treatment was effective for patients with CU. On the other hand, based on the results of subgroup analyses, TG plus H1-AH was superior to H1-AH alone in total efficacy rate at week 2, week 4, and week 8. This result indicated that TG could rapidly improve symptoms of CU, and the synergistic effect of TG was able to persist within 8 weeks as long as it was used. Although the rates between two groups were not statistically different at week 12, the rate of TG plus H1-AH was higher than that of H1-AH alone. The negative result might be attributed to the small number of included patients, and the effect of TG might be underestimated. Therefore, more large-sample studies are needed to draw a more reliable and precise conclusion in the future. Moreover, the total efficacy rates in patients receiving different doses of TG plus H1-AH were significantly higher than those in patients receiving H1-AH alone. This result demonstrated that the low dose of TG (10 - 30 mg/d) could still achieve the effect after 4 weeks of treatment, which might reduce the adverse events and improve the treatment compliance.

Meanwhile, the safety of TG is also important. It is known that the active ingredients of TwHF are also toxic, and the common adverse effects include gastrointestinal discomfort, nausea, elevated levels of liver enzymes, leukopenia, menstrual irregularity and amenorrhea (Ru et al. 2019). However, due to the extraction and purification by modern pharmaceutical technology, the compositions of TG are relatively simple, and some high toxic substances are removed. Therefore, the incidences of adverse effects of TG are remarkably decreased, while its efficacy is retained at the same time. In this meta-analysis, the pooled results showed that there were no statistical differences on the incidences of gastrointestinal discomfort and nausea between two groups, indicating that TG might not significantly increase their occurrences. Other uncomfortable symptoms, such as weakness, dry mouth, drowsiness, dizziness, and headache, might be attributed to the adverse effects of H1-AH. In addition, six and two patients in the TG plus H1-AH groups experienced elevated levels of liver enzymes and amenorrhea, respectively, however, the incidences of two AEs in the TG plus H1-AH groups were not significantly increased. The negative results are in accordance with some previous studies that evaluated the safety of TG for the treatment of rheumatoid arthritis, Sjögren’s syndrome, and diabetic nephropathy. They proved that after 3 to 6 months treatment of TG 30 mg or 60 mg daily, the incidences of liver dysfunction, amenorrhea, and leukopenia in the TG adjuvant therapy groups were not significantly increased in comparison with the control groups (Guo et al. 2021; Luo et al. 2021; Geng et al. 2022). These results demonstrate that the incidences of liver dysfunction, amenorrhea, and leukopenia are low in the patients receiving TG, and the risks of these adverse effects are not remarkably increased along with the accumulated total dose of TG. Therefore, short-term use of TG is a safe treatment for the treatment of CU. In order to early find these adverse effects, regular detection of blood routine test and liver and renal functions at the first month of treatment is necessary, and it could reduce the risk of serious AEs. In addition, because TG could reduce the concentration and survival rate of sperms and make the convoluted meridians vacuolated (Dai et al. 2022), its reproductive toxicity limits the application in some patients at childbearing age, and couples with childbearing demand need to take it cautiously.

However, there are some limitations in this meta-analysis. First, the qualities of the included studies were suboptimal. The detailed information on randomization, allocation concealment, and blinding was missing, and the reliability of evidence was reduced. Second, some outcomes had small numbers of studies and patients, therefore the effect of TG might be underestimated, and the association between efficacy and dosage of TG could not be explored. Third, the treatment durations of all included studies were not beyond 12 weeks, and the efficacy and safety of long-term use of TG for CU remain unclear. Finally, all included studies were conducted in China, and the conclusion might only be applicable for Chinese patients with CU. Efficacy and safety of TG in other countries or races need further investigations. Therefore, more high-quality, large-sample, and long-term trials are required to provide more reliable and accurate evidence in the future.

## Conclusions

This meta-analysis demonstrated that the combination of TG and H1-AH had a good effect on reducing wheals and relieving pruritus in adults with CU. Moreover, short-term use of TG was safe and tolerable, and regular laboratory tests could eliminate the occurrence of serious AEs. However, due to the suboptimal quality of all included studies, more large-scale and high-quality trials are needed to confirm and update the evidence.

## Data Availability

All data generated or analyzed during this study are included in this published article.

## References

[CIT0001] Altman K, Chang C. 2013. Pathogenic intracellular and autoimmune mechanisms in urticaria and angioedema. Clin Rev Allergy Immunol. 45(1):47–62. 22674016 10.1007/s12016-012-8326-y

[CIT0002] Altrichter S, Frischbutter S, Fok JS, Kolkhir P, Jiao Q, Skov PS, Metz M, Church MK, Maurer M. 2020. The role of eosinophils in chronic spontaneous urticaria. J Allergy Clin Immunol. 145(6):1510–1516. 32224275 10.1016/j.jaci.2020.03.005

[CIT0003] Asero R, Tedeschi A, Coppola R, Griffini S, Paparella P, Riboldi P, Marzano AV, Fanoni D, Cugno M. 2007. Activation of the tissue factor pathway of blood coagulation in patients with chronic urticaria. J Allergy Clin Immunol. 119(3):705–710. 17204316 10.1016/j.jaci.2006.08.043

[CIT0004] Chen T. 2017. Study on the effect of *Tripterygium* glycosides tablet combined with desloratadine in the treatment of chronic spontaneous urticaria. Chin Foreign Med Res. 15(7):24–26. Chinese.

[CIT0005] Chen XY. 2016. Assessment of the efficacy of ebastine combined with *Tripterygium* glycosides for chronic urticaria. For All Health. 10(6):145–145. Chinese.

[CIT0006] Chen M, Wang ML, Wang WW. 2017. *Tripterygium* glycosides tablet combined with desloratadine for 42 cases with chronic spontaneous urticaria. Chin J Ethnomed Ethnopharm. 26(20):87–88,95. Chinese.

[CIT0007] Chen Q, Zhong H, Chen WC, Zhai Z, Zhou Z, Song Z, Hao F. 2018. Different expression patterns of plasma Th1-, Th2-, Th17- and Th22-related cytokines correlate with serum autoreactivity and allergen sensitivity in chronic spontaneous urticaria. J Eur Acad Dermatol Venereol. 32(3):441–448. 28846158 10.1111/jdv.14541

[CIT0008] [CSD, CUR] Chinese Society of Dermatology, Centre for Urticaria Research. 2019. [Guideline for diagnosis and treatment of urticaria in China]. Chin J Dermatol. 51(1):1–5. Chinese.

[CIT0009] Dai Y, Sun L, Han S, Xu S, Wang L, Ding Y. 2022. Proteomic study on the reproductive toxicity of *Tripterygium* glycosides in rats. Front Pharmacol. 13:888968. 35668950 10.3389/fphar.2022.888968 PMC9163711

[CIT0010] Dai S, Yin K, Yao X, Zhou L. 2013. Inhibition of interleukin-13 gene expression by triptolide in activated T lymphocytes. Respirology. 18(8):1249–1255. 23796028 10.1111/resp.12145

[CIT0011] Deng M, Liao YM, Liu JH. 2017. The evaluation of *Tripterygium* glycosides tablet combined with desloratadine for the treatment of chronic spontaneous urticaria. Forefront Med. 7(7):7–8. Chinese.

[CIT0012] Fricke J, Ávila G, Keller T, Weller K, Lau S, Maurer M, Zuberbier T, Keil T. 2020. Prevalence of chronic urticaria in children and adults across the globe: systematic review with meta-analysis. Allergy. 75(2):423–432. 31494963 10.1111/all.14037

[CIT0013] Gan MZ, Yu JJ, Deng Y. 2022. Clinical effect of *Tripterygium* glycosides combined with total glucosides of paeony for the treatment of Sjögren’s syndrome and their influences on anticardiolipin antibody and rheumatoid factor. Chin Arch Trad Chin Med. 40:94–96. Chinese.

[CIT0014] Gao P. 2020. Efficacy and safety of ebastine combined with *Tripterygium* glycosides tablet for the treatment of chronic urticaria. J Med Aesthet Cosmetol. 29(13):19–20. Chinese.

[CIT0015] Geng Q, Liu B, Ma Y, Li H, Shi N, Ouyang G, Nie Z, Yi J, Chen Y, Wang Y, et al. 2022. Effects and safety of the *Tripterygium* glycoside adjuvant methotrexate therapy in rheumatoid arthritis: a systematic review and meta-analysis. Evid Based Complement Alternat Med. 2022:1251478. 35368750 10.1155/2022/1251478 PMC8970871

[CIT0016] Gonçalo M, Gimenéz-Arnau A, Al-Ahmad M, Ben-Shoshan M, Bernstein JA, Ensina LF, Fomina D, Galvàn CA, Godse K, Grattan C, et al. 2021. The global burden of chronic urticaria for the patient and society. Br J Dermatol. 184(2):226–236. 32956489 10.1111/bjd.19561

[CIT0017] Guo HB, Peng JQ, Wang X, Zhang KK, Zhong GZ, Chen WH, Shi GX. 2021. Efficacy of *Tripterygium* glycosides for diabetic nephropathy: a meta-analysis of randomized controlled trials. BMC Nephrol. 22(1):304. 34493223 10.1186/s12882-021-02487-8 PMC8425142

[CIT0018] He JX, Cai ZL, Huang WF. 2009. Observation of the effect of combination therapy on chronic spontaneous urticaria. Int Med Health Guid New. 15:76–77,83. Chinese.

[CIT0019] He L, Yi W, Huang X, Long H, Lu Q. 2021. Chronic urticaria: advances in understanding of the disease and clinical management. Clin Rev Allergy Immunol. 61(3):424–448. 34529248 10.1007/s12016-021-08886-x

[CIT0020] He ZS. 2013. Study on the levels of total IgE, C3, C4, IL-2, IL-4, and IFN-γ in serum in the patients with chronic spontaneous urticaria [Dissertation]. Shijiazhuang, Hebei province, Hebei North University.

[CIT0021] Higgins JP, Altman DG, Gøtzsche PC, Jüni P, Moher D, Oxman AD, Savovic J, Schulz KF, Weeks L, Sterne JA, Cochrane Bias Methods Group 2011. The Cochrane Collaboration’s tool for assessing risk of bias in randomised trials. Br Med J. 343(oct18 2):d5928–d5928. 22008217 10.1136/bmj.d5928 PMC3196245

[CIT0022] Li ZK, Wang CZ, Qian GS, Lin KX, Liu G. 2002. Effect of triptolide on expression of IL-5, IL-13 and GM-CSF receptors mRNA in BALF eosinophils of asthmatic guinea pigs. Immunol J. 18:102–106. Chinese.

[CIT0023] Lin KX, Wang CZ, Qian GS. 2000. Effect of triptolide on apoptosis of CD4^+^ and CD8^+^ T cells. Immunol J. 16(1):24–26. Chinese.

[CIT0024] Lin KX, Wang CZ, Qian GS. 2001. Effect of *Tripterygium* glycosides on the production of Th1 and Th2-related cytokines in asthma patients. Chin J Integr Med. 21(1):22–24. Chinese. 12577371

[CIT0025] Liu Z. 2016. Clinical effect of desloratadine combined with *Tripterygium* glycosides tablet on chronic spontaneous urticaria. Clin Med. 36(4):21–22. Chinese.

[CIT0026] Liu L, Zhao H, Sun X, Zheng Q, Luo Y, Ru Y, Zhang Y, Chen X, Zhu B, Yin C, et al. 2018. Efficacy and safety of *Tripterygium wilfordii* Hook F for chronic urticaria: a systematic review and meta-analysis. BMC Complement Altern Med. 18(1):243. 30170584 10.1186/s12906-018-2305-7 PMC6119305

[CIT0027] Long T, Ding ZY, Wang W. 2021. Effect of desloratadine combined with *Tripterygium* glycosides tablet on the clinical efficacy and mood of patients with chronic spontaneous urticaria. J Int Psychiat. 48:1106–1109. Chinese.

[CIT0028] Lu T, Rao YT, Zhang J, Wang YY, Zhang W. 2021. Effect and mechanism of *Tripterygium* glycosides tablet for the treatment of rheumatoid arthritis. J Chin Med Mat. 44:2214–2218. Chinese.

[CIT0029] Luo Y, Zhang Y, Kuai L, Xing M, Ru Y, Luo Y, Liu L, Chen J, Li B, Li X. 2021. Efficacy and safety of *Tripterygium* glycosides in Sjögren’s syndrome treatment: evidence from 12 randomized controlled trials. Ann Palliat Med. 10(7):8215–8231. 34263629 10.21037/apm-21-256

[CIT0030] Matsubara D, Takahagi S, Saito R, Kamegashira A, Tanaka A, Hide M. 2021. Analysis of the long-term economic burden of omalizumab on patients with chronic spontaneous urticaria. J Dermatol. 48(1):56–63. 33029864 10.1111/1346-8138.15630

[CIT0031] Moy AP, Murali M, Nazarian RM. 2016. Identification of a Th2- and Th17-skewed immune phenotype in chronic urticaria with Th22 reduction dependent on autoimmunity and thyroid disease markers. J Cutan Pathol. 43(4):372–378. 26785710 10.1111/cup.12673

[CIT0032] Page MJ, McKenzie JE, Bossuyt PM, Boutron I, Hoffmann TC, Mulrow CD, Shamseer L, Tetzlaff JM, Akl EA, Brennan SE, et al. 2021. The PRISMA 2020 statement: an updated guideline for reporting systematic reviews. Br Med J. 372:n71. 33782057 10.1136/bmj.n71 PMC8005924

[CIT0033] Pei Y. 2017. Clinical observation of combination therapy for the treatment of chronic spontaneous urticaria. J Dermatol Venereol. 39:383–384. Chinese.

[CIT0034] Pereyra-Rodriguez JJ, Galán Gutiérrez M, Armario-Hita JC, Ruiz-Villaverde R. 2020. Prevalence of chronic urticaria refractory to antihistamines in Andalucia, Spain. Dermatol Ther. 33(6):e13866. 32558086 10.1111/dth.13866

[CIT0035] Pi XB, Wang XX, Mai YM, Li JH. 2006. Effect of *Tripterygium* glycosides tablet combined with mizolastine for 22 cases with chronic spontaneous urticaria. South China J Dermato-Venereol. 13:130–132. Chinese.

[CIT0036] Qian M, Zhang SY. 2011. Clinical efficacy and life quality of *Tripterygium* glycosides combined with antihistamine for chronic urticaria. Chin J Dermatovenerol Integr Trad Western Med. 10:359–362. Chinese.

[CIT0037] Qin ZM, Han XM. 2017. Analysis of the efficacy of ebastine combined with *Tripterygium* glycosides for the treatment of chronic urticaria. J Clin Med. 4:17396–17396. Chinese.

[CIT0038] Ren Y, Zhang JL, Yang ZY. 2022. Effect of Biqi capsule combined with *Tripterygium* glycoside in the treatment of ankylosing spondylitis and its influence on blood hypercoagulable state and B and T lymphocyte attenuator. Clin Res Pract. 7:87–89. Chinese.

[CIT0039] Ru Y, Luo Y, Zhou Y, Kuai L, Sun X, Xing M, Liu L, Lu Y, Hong S, Chen X, et al. 2019. Adverse events associated with treatment of *Tripterygium wilfordii* Hook F: a quantitative evidence synthesis. Front Pharmacol. 10:1250. 31780926 10.3389/fphar.2019.01250 PMC6851843

[CIT0040] Shi YS, Wang QM, Qiu XJ, Pang GX. 2019. Meta-analysis of efficacy and safety of *Tripterygium* glycosides tablets combined with desloratadine in treatment of chronic urticarial. Zhongguo Zhong Yao Za Zhi. 44(16):3551–3557. Chinese 31602921 10.19540/j.cnki.cjcmm.20181112.001

[CIT0041] Takahagi S, Mihara S, Iwamoto K, Morioke S, Okabe T, Kameyoshi Y, Hide M. 2010. Coagulation/fibrinolysis and inflammation markers are associated with disease activity in patients with chronic urticaria. Allergy. 65(5):649–656. 19845571 10.1111/j.1398-9995.2009.02222.x

[CIT0042] Wang D. 2010. Observation of effect of ebastine combined with *Tripterygium* glycosides for the treatment of chronic urticaria. Med Inf. 23:4215–4217. Chinese.

[CIT0043] Wang Q. 2014. Observation of the efficacy of *Tripterygium* glycosides tablet combined with desloratadine for chronic spontaneous urticaria. Chin J Mod Drug Appl. 8(24):98–99. Chinese.

[CIT0044] Wang Y. 2018. Clinical observation of ebastine combined with *Tripterygium* glycosides for the treatment of chronic urticaria. Home Med. (12):176–177. Chinese.

[CIT0045] Wei YH, Zhang JH, An RZ, Wang JL. 2010. Combination effect of *Tripterygium wilfordii* with desloratadine on the treatment of chronic idiopathic urticaria. Chin J Derm Venereol. 24:1170–1172. Chinese.

[CIT0046] Xiao CC, Zhang CY. 2019. Clinical observation of setastine combined with *Tripterygium* glycosides tablet for the treatment of chronic urticaria. Chin J New Drugs. 28:2857–2859. Chinese.

[CIT0047] Xie YL, Mu SY, Zhao XF, Mu SL. 2012. Clinical effect of *Tripterygium* glycosides tablet combined with desloratadine for chronic spontaneous urticaria. China Rural Health. (z1):80–81. Chinese.

[CIT0048] Xing BF, Feng LX. 2017. Clinical study of desloratadine and *Tripterygium wilfordii* tablets in the treatment of chronic idiopathic urticaria. Chin Community Doct. 33(15):70,72. Chinese.

[CIT0049] Xu SX. 2018. Evaluation of the effect of ebastine combined with *Tripterygium* glycosides for chronic urticaria. For All Health. 12(2):161–162. Chinese.

[CIT0050] Yang HJ. 2019. Clinical efficacy of *Tripterygium* glycoside combined with desloratadine for chronic urticaria. J Med Aesthet Cosmetol. 28(2):77–77. Chinese.

[CIT0051] Yang L. 2011. Clinical observation of *Tripterygium* glycosides tablet combined with desloratadine for the treatment of chronic spontaneous urticaria. J China Trad Chin Med Inf. 3(17):130–130. Chinese.

[CIT0052] Yang ZM, Cheng JF, Chen MD, Liang YB. 2015. Effect of triptolide on IL-23/Th17(IL-17) inflammatory axis in asthmatic BALB/c mice. Chin J Immunol. 31:1347–1351,1356. Chinese

[CIT0053] Yao CY, Wang T, Jiang YL, Zheng X, Lin PH. 2017. Effect of celastrol on mast cell and PI3K/AKT/GSK3-ß pathway. Fujian Med J. 39(1):61–64. Chinese.

[CIT0054] Zhang BY. 2021. Efficacy of *Tripterygium* glycosides combined with secondary H1-antihistamine for the treatment of chronic urticaria. Contemp Med Symp. 19(10):145–146. Chinese.

[CIT0055] Zhang XJ, Ma Y. 2021. Efficacy and safety of ebastine combined with *Tripterygium* glycosides tablet for chronic urticaria. J North Pharm. 18(5):119–120. Chinese.

[CIT0056] Zhang X, Song X, Zhang M, Li C, Huang Z, Liu B, Yu M, Liao S, Luan T, Zuberbier T, et al. 2022. Prevalence and risk factors of chronic urticaria in China: a nationwide cross-sectional study. Allergy. 77(7):2233–2236. 35332543 10.1111/all.15287

[CIT0057] Zhang WY, Wang T, Xu NH, Chen JY. 2018. Celastrol down-regulate the NK-1R mRNA expression of mast cell mediated by substance P. Fujian Med J. 40(1):120–123. Chinese.

[CIT0058] Zhao RY. 2018. Efficacy of *Tripterygium* glycosides tablet combined with desloratadine for the treatment of chronic spontaneous urticaria. Health Must-Read Mag. (18):109–109. Chinese.

[CIT0059] Zhong JX, Yan HF, Huo KM, Gu YN, Huang XX, Zhuang XJ, Chen HB. 2020. The effect of gamma globulin combined with *Tripterygium wilfordii* glycoside on children with Henoch-Schonlein purpura nephritis. Chin J Diffic Complicat Cases. 19:66–70. Chinese.

[CIT0060] Zhou ZD. 2014. Analysis of the efficacy of *Tripterygium* glycosides tablet combined with desloratadine for the treatment of chronic spontaneous urticaria. Med Inf. 27(11):304–305. Chinese.

[CIT0061] Zhou ML, Lu MH, Liu JP, Liu LL, Yin BH, Gao SQ. 2018. Effect of *Tripterygium* glycosides tablet on the serum levels of endostatin and TSP-1 in patients with chronic spontaneous urticaria. Lab Med Clinic. 15:973–975. Chinese.

[CIT0062] Zhu XL. 2018. Effect of desloratadine combined with *Tripterygium* glycosides tablet in the treatment of chronic spontaneous urticaria. Home Med. (9):58–59. Chinese.

[CIT0063] Zuberbier T, Abdul Latiff AH, Abuzakouk M, Aquilina S, Asero R, Baker D, Ballmer-Weber B, Bangert C, Ben-Shoshan M, Bernstein JA, et al. 2022. The international EAACI/GA^2^LEN/EuroGuiDerm/APAAACI guideline for the definition, classification, diagnosis, and management of urticaria. Allergy. 77(3):734–766. 34536239 10.1111/all.15090

